# Proportion and predictors of transfusion-transmissible infections among blood donors in North Shewa Zone, Central North Ethiopia

**DOI:** 10.1371/journal.pone.0194083

**Published:** 2018-03-26

**Authors:** Tekalign Deressa, Wubet Birhan, Bamlaku Enawgaw, Molla Abebe, Habtamu Wondiferaw Baynes, Mekuria Desta, Betelihem Terefe, Mulugeta Melku

**Affiliations:** 1 School of Biomedical and Laboratory Sciences, College of Medicine and Health Sciences, University of Gondar, Gondar, Ethiopia; 2 Ethiopian Public Health Institute, Addis Ababa, Ethiopia; 3 Debre Berhan Blood Bank, North Shoa Zone, Debre Berhan, Ethiopia; University of Cincinnati College of Medicine, UNITED STATES

## Abstract

**Background:**

Transfusion-transmissible infections (TTIs) pose a significant challenge for the availability and safety of blood transfusion. The aim of this study was to investigate the prevalence and risk factors for TTIs among blood donors in North Shewa zone, central North Ethiopia.

**Methods:**

A retrospective survey of blood donors’ medical records was conducted from April 2014 to June 2017 to assess the presence of hepatitis B virus (HBV), hepatitis C virus (HCV), human immunodeficiency virus (HIV), and syphilis infections. Descriptive statistics such as percentage, median and interquartile range were used to summarize the data.

**Results:**

Out of 8460 donations, 207 (2.4%, 95% CI 2.06–2.71%) had serological evidence of infection with at least one pathogen. Four of the blood donors (0.047%) had co-infection with more than one pathogen; 2HIV/HBV and 2HIV/syphilis. The overall prevalence of HBV, HCV, HIV, and syphilis among the donors were 1.2% (95% CI 0.98–1.45%), 0.32% (95% CI 0.2–0.44%), 0.25% (95% CI 0.14–0.35%), and 0.71% (95% CI 0.53–0.89%) respectively. Male sex was significantly associated with higher risk of HBV (OR 1.75, 95% CI 1.1–2.8) and syphilis sero-reactivity (OR 4.5, 95% CI1.9–10.5). Farmers and older donors were found to be at a higher risk for syphilis seropositivity.

**Conclusion:**

The prevalence of TTIs among blood donors in North Shewa zone was relatively low compared to those of other geographic places in Ethiopia. However, TTIs remain a concern for the availability and safety of blood transfusion as they are still prevalent in the study area. Therefore, more efforts are required to ensure the safety of blood supply and transfusions.

## Introduction

Blood transfusion is an essential part of any strong health system and saves millions of lives worldwide each year [1]. However, it is also a potential source for transmission of several life-threatening pathogens. These including human immunodeficiency virus (HIV), hepatitis B virus (HBV), hepatitis C virus (HCV), West Nile virus, and a myriad of bacterial and protozoal pathogens [2, 3]. The risk for acquiring transfusion-transmissible infections (TTIs) in blood donation has been significantly reduced in developed countries [1–4]. However, there are still high risks of TTIs in resource-limited settings such as sub-Sahara Africa due to inadequacies of screening facilities and qualified personnel [3, 4]. According to recent estimates, for example, blood transfusion has been accounted for 5–10% of HIV infections and 4–12.5% post-transfusion hepatitis in sub-Saharan Africa [5].

Ethiopia is one of the low-income countries with a high burden of TTIs. The prevalence of TTIs in Ethiopia shows a marked heterogeneity across sub-group of the population, geographic areas and periods of studies [6–10]. For instance, a study conducted in Northwest Ethiopia among blood donors in 2010 showed that the prevalence of HIV, HBV, HCV, and syphilis was 3.8%, 4.7%, 0.7%, and 1.3% respectively [6]. A similar study conducted in Eastern Ethiopia in 2016 estimated the overall seroprevalence of HIV, HBV, HCV, and syphilis at 3.16%, 9.48%, 0.73% and 0.73% respectively [7]. Furthermore, according to a recent meta-analysis report, an overall pooled prevalence of HBV and HCV among Ethiopian population was 7.4% (95%CI: 6.5–8.4) and 3.1% (95%CI: 2.2–4.4) respectively. The pooled prevalence of HBV among blood donors was 8.4% (95%CI: 5.4–12.7) [8]. These reports indicated that TTIs pose a significant threat to blood safety, and causing critical concerns to blood transfusion programs in Ethiopia.

To ensure the safety of blood supply, the government of Ethiopia has -implemented careful donor selection criteria and quality assured screening of all donated blood for major TTIs, including HBV, HCV, HIV, and syphilis prior to clinical use. Furthermore, the country has implemented a number of programs to reduce the burden of blood-borne pathogens among its population. These include, but not limited to, public awareness creation on transmission and prevention of blood-borne infection, free HIV testing and counseling, antenatal screening of pregnant women for syphilis, HIV and HBV, immunization of children and at-risk groups such as health workers against HBV, and universal access to HIV antiviral therapy [11–13].

Continuous monitoring of the prevalence of TTIs among blood donors provides valuable information for improving blood safety, availability and the quality of transfusion service. Studies from different parts of Ethiopia had reflected the critical challenge of TTIs for safe blood supply [6–12]. However, data on the prevalence rate of these infections among the donor populations in North Shewa Zone blood bank, central North Ethiopia, is lacking. North Shewa Zone blood bank is one of the newly established blood banks in 2014 under the National Blood Transfusion Service Agency [13]. Most of the donor populations in this area are from small towns and rural districts. Consequently, the epidemiology of TTIs in this area could be different from those reported from other parts of the country. Therefore, this study was conducted to report the prevalence and associated factors for HBV, HCV, HIV and *T*. *pallidum* infections among blood donors in this part of Ethiopia, from March 2014 through June 2017.

## Materials and methods

### Study design, setting and population

**Fig 1:**
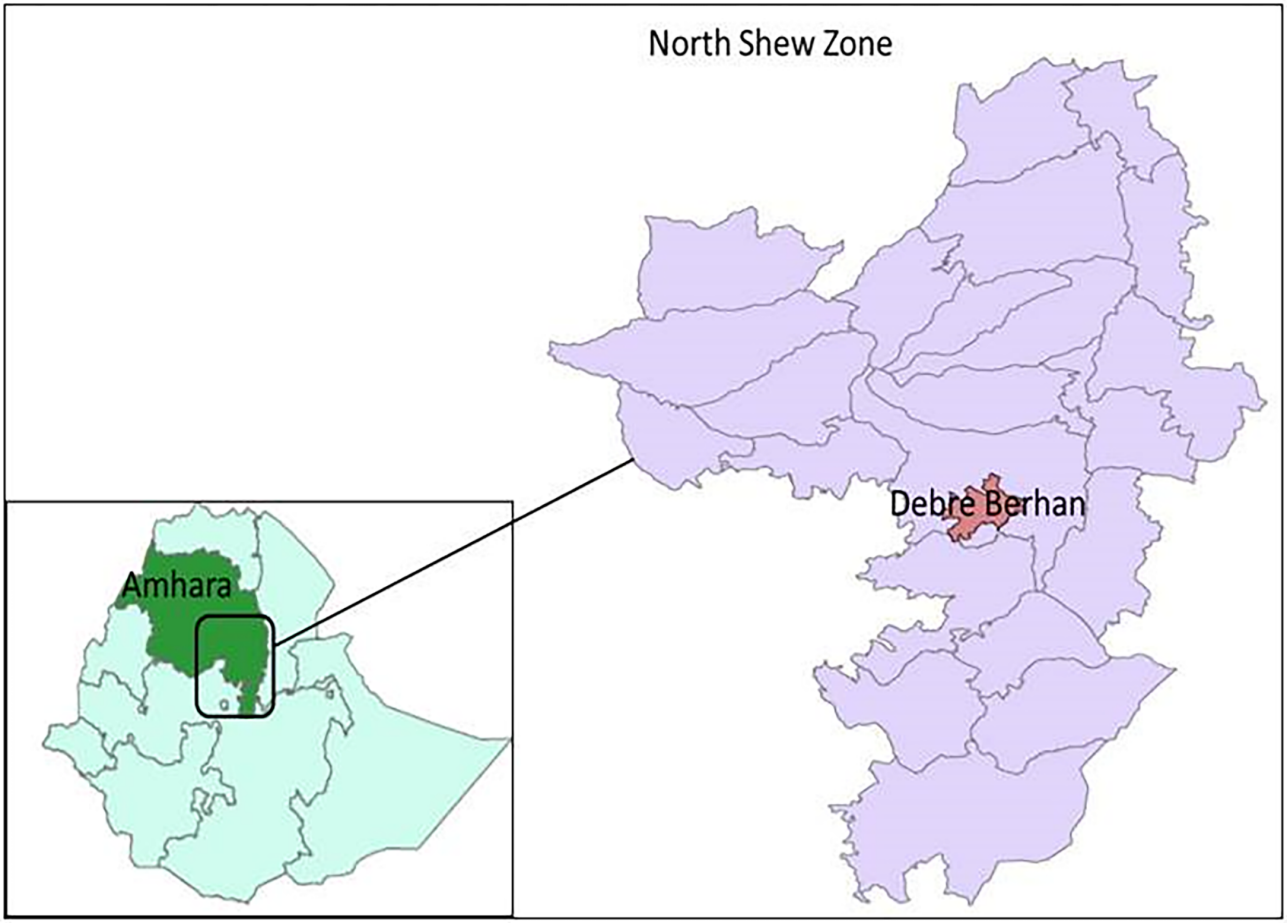
Map of North Shewa zone (Right) and Ethiopia (Left).

This retrospective study was conducted on blood donors data recorded at North Shewa Zone blood Bank, Central North Ethiopia, from March 2014 to June 2017 (Fig 1). North Shewa is one of 10 Zones in the Amhara Regional state. It has 24 districts, of which 3 urban and 21 rural districts. According to the 2007 Population and Housing Census of Ethiopia, the population of North Shewa Zone was about 1.9million. The major ethnic groups of the zone are Amhara and over 94% of the population is Orthodox-Christian by religion. The study area included two parts, Debre Berhan town, and surrounding districts. Debre Berhan center is the largest center that contributes to collecting most of the blood units. All blood donors were apparently healthy non-remunerated volunteers.

### Data collection

All blood donors went through a health history interview and physical examination for eligibility according to the national blood donation criteria. The eligibility criteria were age between 17 and 65 years, body weight >45 kg, physical fitness, no history of high-risk sexual behavior and practice, blood transfusion, jaundice, hepatitis, surgery, and hypertension, and current fever. Venous blood was collected from each donor by laboratory technologists following standard procedures.

### Laboratory methods

All donated blood samples were screened for HIV-1 and 2 using a Vironostika HIV Uni-Form II Ag/Ab fourth generation ELISA (Bio-Merieux, Boxtel, Netherlands). Syphilis seropositivity was tested by using rapid plasma reagin (RPR) (Omega Immutrep-RPR®, UK) following the manufacturer’s instructions. Reactive samples to RPR were confirmed by using *Treponema pallidum* hemagglutination assay (TPHA) (Omega Immutrep TPHA®, UK). The presence of hepatitis B surface antigen (HBsAg) (Hepanostika HBsAg UNi-Form II, Bio-Merieux, Boxtel, Netherlands) and anti-HCV antibody (HumanGasellschaft for Biochemical and diagnostic GMbH, Germany) were determined using ELISA technique according to the manufacturer’s instruction. The sensitivity and specificity of each assay are presented in S1 Table.

### Statistical analysis

Data were entered, cleaned and analysed using SPSS statistical software (SPSS Inc., Chicago, IL, USA). The results were stratified by gender, age, occupation and the year of donations. Descriptive data were presented as frequencies and percentages. Chi-square test was used to compare the seroprevalence rates of TTIs among blood donors grouped according to socio-demographic characteristics and to analyze the variations in trends of TTIs during the study period. Bivariate and multivariable binary logistic regression models were fitted to identify factors associated with TTIs. The odds ratio and its 95% confidence interval (CI) were used to determine the strength of the association. Statistical significance was set at P values of less than 0.05.

### Ethics statement

This study was approved by the institutional ethics review board of the University of Gondar (IRB Ref No: O/V/P/RCS/05/479/2015). However, informed consent was not obtained from the study participants due to the nature of the study (retrospective chart review). All records of blood donors were de-identified and analysed anonymously.

## Results

### Demographic characteristics of the blood donors

A total of 8460 consecutive donors’ blood was screened for four TTIs at North Shewa zone Blood bank from March 2014 through June 2017. Most of the donors were male 5644 out of 8460 (66.7%), aged between 18–26 years (71.9%). The median age of the donors was 22 year (range: 18–65). A little over half 4581/8460(54.1%) of the blood donors were students followed by government employees 2679/8460(31.7%) (Table 1). First-time donors (over 98%) represented the majority of blood donors at the study area.

**Table 1:** Demographic characteristics of blood donors at Debre Berhan blood bank, Central North Ethiopia, March 2014-June 2017.

Variables		Frequency (N)	Percent (%)
**Sex**	Male	5644	66.7
	Female	2816	33.3
**Age (Years)**	18–20	3096	36.6
	21–26	2983	35.3
	27–40	1970	23.3
	41–65	411	4.9
**Occupation**	Student	4581	54.1
	Gov. employee	2679	31.7
	Private Employee	686	8.1
	Daily laborer	108	1.3
	Farmer	406	4.8
**Site**	Debre Berhan	6868	81.2
	Others	1592	18.8
**Year**	2014	1033	12.2
	2015	3342	39.5
	2016	2338	27.6
	2017	1747	20.7
**Overall**		8460	100

### Prevalence of transfusion-transmissible infections and risk factors

Out of all blood donations screened for TTIs during the study period, 207 (2.4%, 95% CI 2.06–2.71%) had serological evidence of infection with at least one pathogen. Four of the blood donors (0.047%) had co-infection with more than one pathogen; two with HIV/HBV and two with HIV/syphilis. Hepatitis B virus was the most prevalent TTI (1.2%; 95% CI 0.98–1.45%) among the blood donors. The overall prevalence rates of HCV, HIV, and syphilis among the donors were 0.32% (95% CI 0.2–0.44%), 0.25% (95% CI 0.14–0.35%), and 0.71% (95% CI 0.53–0.89%) respectively (Table 2).

**Table 2:** Positivity rate of transfusion-transmissible infections with occupation among blood donors in North Shewa Zone, Central North Ethiopia, 2014–2017.

Occupation	Donation (n)	Seropositivity, n(%)				Overall Positivity
		HBsAg	HCV	HIV	Syphilis	
Student	4581	54(1.2)	15(0.3)	10(0.2)	21(0.5)	100(2.2)
Gov. employee	2679	41(1.5)	10(0.4)	9(0.3)	24(0.9)	84(3.1)
Private Employee	686	2(0.3)	0(0.0)	1(0.1)	4(0.6)	7(1.0)
Daily laborer	108	2(1.9)	0(0.0)	1(0.9)	0(0.0)	3(2.8)
Farmer	406	4(1.0)	2(0.5)	0(0.0)	11(2.7)	17(4.2)
p-value		0.14	-	-	-	
95% CI		0.98–1.45	0.2–0.44	0.14–0.35	0.53–0.89	2.06–2.71
Total	8460	103(1.2)	27(0.32)	21(0.25)	60(0.71)	207(2.4)

HBsAg: Hepatitis B surface antigen, HCV: Hepatitis C virus, HIV: human immunodeficiency virus

In terms of the donors’ occupation, the highest seroprevalence of TTIs was observed among farmers. But, the lowest sero-prevalence (1.0%) was noted among private workers (Table 2). Of the four TTIs screened, only HBV was observed in all occupation categories, with the highest seroprevalence amongst daily laborers 1.9% (COR 0.63; 95%CI: 0.2–2.6). The seroprevalence of syphilis (2.7%) was higher among farmers compared to other occupations.

**Fig 2:**
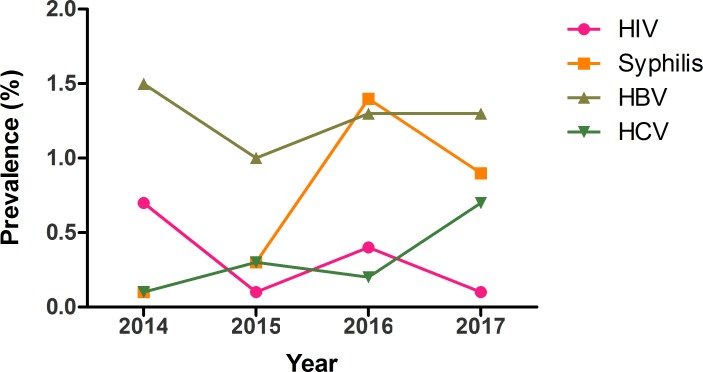
Yearly prevalence of transfusion-transmissible infections among blood donors in North Shewa Zone, central North Ethiopia.

The yearly prevalence rates of HBV, HCV, HIV, and syphilis are depicted in Fig 2. The overall yearly prevalence of the four TTIs was 2.3% (24/1033), 1.7% (58/3342), 3.3% (77/2338) and 3.0% (52/1747) in the year 2014, 2015, 2016 and 2017 respectively. Significant increases were noted in the prevalence of syphilis infections from 2014 to 2016 (P = 0.008).

The prevalence of the four TTIs varied according to the demography of the blood donors. The seroprevalence of HBV, HCV, HIV and syphilis among male donors were 1.4% (80/5644), 0.3% (18/5644), 0.2% (13/5644), and 1.0% (54/5644) respectively. The seroprevalence of HBV and HCV (Table 3), and HIV and syphilis (Table 4) among female donors were 0.8% (23/2816), 0.3% (9/2816), 0.3% (8/2816), and 0.2% (6/2816) respectively. The difference in seropositivity of HBV (OR 1.75; 95%CI: 1.1–2.8) and syphilis (OR 4.5; 95%CI: 1.9–10.5) between male and female donors were statistically significant. With respect to ages of the donors, the highest and the lowest seroprevalence of HBV (1.6% vs 0.7%) was noted among donors aged between 27-40years and 41–65 years respectively (p>0.05). While the highest and lowest seroprevalence of Syphilis (3.6% vs 0.3%) was observed among the donors in the age group of 41–65 years and 21–26 years respectively (P<0.001). The prevalence of syphilis increased with the age of donors (Table 4).

**Table 3:** Seroprevalence of HBVand HCV infections according to socio-demographic characteristics of blood donors at Debre Berhan Blood Bank, Central North Ethiopia.

Variables	HBV+	COR(95% CI)	P-value	HCV+	COR(95% CI)	P-value
	n(%)			N(%)		
**Sex**						
Male	80(1.4)	1.75 (1.1–2.8)	0.01	18(0.3)	0.9(0.4–2.2)	0.9
Female	23(0.8)	1.0	-	9(0.3)	1.0	-
**Age (Years)**						
18–20	34(1.1)	0.66 (0.2–2.2)	0.49	8(0.3)	1.9(0.4–9.0)	0.42
21–26	35(1.2)	0.61(0.2–2.0)	0.42	11(0.4)	1.3(0.3–6.0)	0.72
27–40	31(1.6)	0.46(0.1–1.5)	0.20	6(0.3)	1.6(0.3–8.0)	0.57
41–65	3(0.7)	1.0	-	2(0.5)	1.0	-
**Occupation**						
Student	54(1.2)	1.0	-		-	-
Gov. employee	41(1.5)	0.77 (0.5–1.2)	0.21		-	-
Private Employee	2(0.3)	4.1(1.0–16.0)	0.05		-	-
Daily laborer	2(1.9)	0.63(0.2–2.6)	0.53		-	-
Farmer	4(1.0)	1.2(0.4–3.3)	0.72		-	-
**Year of Donation**						
2014	15(1.5)	0.9(0.5–1.7)	0.77	1(0.1)	7.1(0.9–54.9)	0.06
2015	35(1.0)	1.3(0.7–2.1)	0.39	9(0.3)	2.6(1.1–6.1)	0.03
2016	30(1.3)	1.0(0.6–1.8)	0.92	5(0.2)	3.2(1.1–9.2)	0.03
2017	23(1.3)	1.0	-	12(0.7)	1.0	-

HBV: hepatitis B virus, HCV: Hepatitis C virus, COR: crude odds ratio, CI: confidence intervals

**Table 4:** Sero-prevalence of HIVand syphilis infections according to socio-demographic characteristics of blood donors at Debre Berhan Blood Bank, Central North Ethiopia.

Variables	HIV+	COR(95% CI)	P-value	Syphilis+	COR(95% CI)	P-value
	n(%)			N(%)		
**Sex**						
Male	12(0.2)	1.0	-	54(1.0)	1.0	-
Female	8(0.3)	0.8(0.3–1.9)	0.64	6(0.2)	4.5(1.9–10.5)	<0.001
**Age (Years)**						
18–20	5(0.2)	4.6(1.1–19.1)	0.03	13(0.4)	9.0(4.2–19.0)	<0.001
21–26	8(0.3)	2.7(0.7–10.3)	0.14	8(0.3)	14(5.9–33.4)	<0.001
27–40	5(0.3)	2.9(0.7–12.1)	0.15	24(1.2)	3.1(1.6–5.9)	0.001
41–65	3(0.7)	1.0	-	15(3.6)	1.0	-
**Year of Donation**						
2014	7(0.7)	1.0	-	1(0.1)	1.0	-
2015	4(0.1)	5.7(1.7–19.5)	0.006	10(1.3)	0.3(0.04–2.5)	0.28
2016	9(0.4)	1.8(0.7–4.8)	0.26	33(1.4)	0.1(0.01–0.5)	0.008
2017	1(0.1)	11.9(1.5–96.9)	0.02	16(0.9)	0.1(0.01–0.8)	0.029

HIV: human immunodeficiency virus, COR: crudes odds ratio, CI: confidence interval

### Risk factors for TTIs seropositivity

Bivariate and multivariable logistic regression analyses were performed to identify risk factors for TTIs among blood donors. Variables like age, sex, occupation, years of donation were included in the analysis. Male sex was significantly associated with higher risk of HBV (OR 1.75, 95% CI 1.1–2.8) and syphilis sero-reactivity (OR 4.5, 95% CI1.9–10.5) (Table 3). Donors between the ages of 18–20 years were least likely to be infected by HIV (OR 4.6; 95%CI 1.1–19.1) and syphilis (OR 9.0; 95%CI 4.2–19.0) compared to the highest age group (Table 4). In multivariable logistic regression analysis, age and sex remained significant and independent predictors of syphilis seropositivity. But, none of the variables included into this study were significantly associated with HCV infection.

## Discussion

Safe blood supply is an essential component in improving health care. However, the risk of TTIs remains a critical concern for the safety of blood transfusion programs in resource-limited countries [14–16]. Thus, up-to-date data on the proportion of TTIs among blood donors, especially in TTIs high burden settings like Ethiopia, would inform policy makers to formulate strategies to plan, implement, and evaluate performances. To our knowledge, this is the first comprehensive study on the epidemiology of TTIs among blood donors in North Shewa Zone, Central North Ethiopia.

In this study, we found that 2.4% of the donated blood was tested seropositive for at least one of the screened TTIs. This was relatively a low prevalence when compared to the rates reported from other parts of Ethiopia, which ranged from 6.6% to 29.5% [7, 9, 15, 16]. Several explanations can be put forward for the lower prevalence of TTIs in this study. First, all of the blood donors in our study were voluntary donors, while in the previous studies over 90% of donations were by replacement donors (i.e., a friend or family member of the recipient who donates to replace the stored blood used in a transfusion) [7, 15, 16]. Second, over half of the donors in our study were students and about 40% were employed, unlike other studies [10, 15]. These suggest that the blood donors in the study area might be well aware of their health conditions in terms of TTIs, and it is likely that they might have better awareness on transmission routes and preventions of these infections. Third, it could also relate to variations in donors selection procedure, socioeconomic status, lifestyle and level of awareness between different regions of the country. Yet, the rate found in this study is higher when compared with the prevalence rates from other developing and developed countries [17, 18]. This data suggesting that there is still a need for stringent donor selection and blood screening measures to improve the safety of blood supply.

In this study, there was no obvious increasing or decreasing trend of TTIs during 2014–2017. This could be due to a short study period for trend analysis and due to a relatively low prevalence of the TTIs in the study area.

We found that HBV was the most prevalent TTIs in the study area with 1.2% of the donated blood tested positive for HBsAg. This was relatively a low prevalence rate when compared to 9.5%, 3.6%-6.2%, and 9.48% rates from south [9], Northwest [6, 10, 19], and East Ethiopia [20] respectively. These differences in the prevalence of HBV infection across the studies might be due to some differences in socioeconomic status, risk behaviors, the rate of HBV infection in the background population, and effectiveness of donor selection strategies. It is also important mentioning that our study analysed data collected over three years unlike some of the studies that looked for the prevalence of TTIs over a short period (about a year) [9, 16, 19]. Thus, the observed variations in TTIs prevalence across the studies could be due to such differences. Our study also found that HBV prevalence was higher among male donors than females. This was in agreement with a number of previous studies, although reasons for such gender disparity are unclear [17, 18, 21]. Gender differences in behavioral risk factors such as having multiple sex partners could explain the finding higher prevalence of HBV infection in male donors.

The overall seroprevalence of HCV among the donors was 0.32% (95% CI 0.2–0.44%). This study also revealed that there were no significant differences in HCV infection with respect to the demographic characteristics of the donors possibly due to the overall low rate of HCV. When compared with previous studies from other parts of Ethiopia, the finding 0.32% prevalence rate was lower than those from Gondar (0.7%-5.8%), Jigjiga (0.73%) and Wolayta Sodo (8.5%) [6, 7, 9, 10]. It was also lower than those of the other countries such as Nigeria (6.0%), Tanzania (8.0%), and Equatorial Guinea (3.71%) [22–24]. The variation in the prevalence of HCV infections across studies might be due to differences in risk behaviors such as injecting drug use and sharing objects for skin piercing between different geographical areas.

The 0.25% prevalence rate of HIV among blood donors in North Shewa zone was lower than the previous reports from Gondar (2.24%), Jimma (2.1%), Hawassa (1.6%) and Wolayita Sodo (6.4%), Ethiopia [9, 15, 16, 25]. This could be due to differences in sociodemographic risks factors, HIV prevalence in the general population, health education programs, and donor selection schemes across the study areas. Our study did not find a significant difference in HIV prevalence with the gender of the donors. This was in accordance with some of the previous studies [18, 20, 26]. We also noted that younger donors (18–20 years) were the least affected group by HIV infection, which was inconsistent with a number of previous reports [25–27]. The discrepancy between our finding and others could be attributed to the fact that most of the donors between the ages of 18–20 years in our study were university students who might be well aware of their HIV status.

The seroprevalence of syphilis (0.71%) found in this study was higher when compared to 0.3% among blood donors in Namibia, 0.49% among Eritrean donors and 0.0% among Iranian donors [17, 28, 29]. Nevertheless, it is low compared to 21.5% reported by Xie *et al*. among Equatorial Guinea blood donors, 7.5% by Adjei *et al*. among Ghanaian blood donors, and 4.9% by Buseri *et al*. among Tanzanian donors [22, 23, 30]. Our study also reflected the relatively low prevalence of syphilis in the study area when compared to reports from other regions in the country [7, 9, 10]. Consistent with the previous studies, our data revealed that males, older donors, and farmers were at higher risk for syphilis seropositivity [17, 18, 21]. This could be attributed to the prevalent risk behaviors such as having multiple sex partners and alcohol abuse among males than females. The relatively higher syphilis seropositivity among farmers and older donors might be related to low awareness of TTIs due to their low education levels. Of note, the prevalence of syphilis showed an increasing trend. Apart from this, our study revealed that farmers carry the highest proportion of TTIs compared to those in other occupations. Therefore, concerted efforts that target these groups are required to minimize the risk of syphilis as well as other TTIs in the general population, and to reduce their potential risk to the blood supply.

This study has some limitations in that it analysed the records of blood donors that have not been verified independently. There are no molecular data for any of the pathogens evaluated. Therefore, the accuracy of this data depends on the data collection, recording, and screening systems of the blood bank. Furthermore, it could not include all variables that might associate with TTIs due to its retrospective nature. Despite the limitations, this study highlighted the proportion and risk factors of major TTIs in the area.

In conclusion, the prevalence of TTIs among blood donors in North Shewa zone was found to be low compared to those of other geographic places in Ethiopia. Nevertheless, TTIs remain a concern for the safety of blood supply as they are still prevalent among the blood donors in the area. Therefore, more efforts are required to ensure the safety of blood supply and transfusions.

## Supporting information

S1 Table Sensitivity and specificity of the assays used for screening donated blood. (DOCX)
