# Supplementary material for: Proportion and predictors of transfusion-transmissible infections among blood donors in North Shewa Zone, Central North Ethiopia
Source: PLoS One. 2018 Mar 26;13(3):e0194083. doi: 10.1371/journal.pone.0194083 (PMC5868787; doi:10.1371/journal.pone.0194083)
Supplement: S1 Table — (DOCX) [file pone.0194083.s001.docx]

| **Diagnostic kits** | **Sensitivity** | **Specificity** |
| --- | --- | --- |
| Immunotrep-RPR | 100% | Not indicated |
| Immunotrep-TPHA | 98.5% | 99.6% |
| Hepanostika HBsAg UNi-Form II | 100% | 99.9% |
| Vironostika HIV Uni-Form II Ag/Ab | >98% | 99.9% |
| Anti-HCV | 100% | 99.7% |

**S1 Table. Sensitivities and specificities of the assays used for screening donated blood at North Shewa Zone blood bank, Central North Erhiopia**
